# Comparative Performance Assessment of Novel Fluorescence Immunoassay POCTs for Measuring Circulating Levels of Vitamin-D

**DOI:** 10.3390/molecules29071636

**Published:** 2024-04-05

**Authors:** Alice Palermiti, Alessandra Manca, Fabrizio Mastrantonio, Domenico Maiese, Aurora Curatolo, Miriam Antonucci, Marco Simiele, Amedeo De Nicolò, Antonio D’Avolio

**Affiliations:** 1 Laboratory of Clinical Pharmacology and Pharmacogenetics, Department of Medical Sciences, University of Turin, 10149 Turin, Italyamedeo.denicolo@unito.it (A.D.N.);; 2 A. Menarini Diagnostics, 50131 Florence, Italy; 3 CoQua Lab s.r.l., 10149 Turin, Italy; domaiese08@gmail.com (D.M.); aurora.curatolo98@gmail.com (A.C.);

**Keywords:** 25-OH-Vitamin D, quantitation technique, point-of-care test

## Abstract

Vitamin D (Vit D) is a fat-soluble molecule acting like a hormone, and it is involved in several biological mechanisms such as gene expression, calcium homeostasis, bone metabolism, immune modulation, viral protection, and neuromuscular functions. Vit D deficiency can lead to chronic hypocalcemia, hyperparathyroidism, and many other pathological conditions; in this context, low and very low levels of 25-hydroxy-vitamin D (25-OH-D) were found to be associated with an increased risk of COVID-19 infection and the likelihood of many severe diseases. For all these reasons, it is important to quantify and monitor 25-OH-D levels to ensure that the serum/blood concentrations are not clinically suboptimal. Serum concentration of 25-OH-D is currently the main indicator of Vit D status, and it is currently performed by different assays, but the most common quantitation techniques involve immunometric methods or chromatography. Nevertheless, other quantitation techniques and instruments are now emerging, such as AFIAS-1^®^ and AFIAS-10^®^ (Boditech and Menarini) based on the immunofluorescence analyzer, that guarantee an automated system with cartridges able to give quick and reliable results as a point-of-care test (POCT). This work aims to compare AFIAS-1^®^ and AFIAS-10^®^ (Boditech and Menarini) Vit D quantitation with Ultra High-Performance Liquid Chromatography coupled with tandem mass spectrometry that currently represents the gold standard technique for Vit D quantitation. The analyses were performed in parallel on 56 samples and in different conditions (from fresh and frozen plasma) to assess the reliability of the results. Any statistically significant differences in methods, the fixed error, and the error proportional to concentration were reported. Results obtained in all conditions showed a good correlation between both AFIAS^®^ instruments and LC-MS/MS, and we can affirm that AFIAS-1^®^ and AFIAS-10^®^ are reliable instruments for measuring 25-OH-D with accuracy and in a fast manner.

## 1. Introduction

AFIAS^®^ Vitamin D Neo is a fluorescence immunoassay (FIA) for the quantitative determination of total 25-OH Vitamin D (D2/D3) levels in human whole blood/serum/plasma. It runs on the AFIAS^®^ range of automatic immunofluorescence analyzers (Boditech and Menarini), particularly on AFIAS-1^®^, ideal for street pharmacies, doctor’s office, and hospital bed-side testing, and on AFIAS-10^®^, specifically conceived for satellite labs, small routine, and other decentralized scenarios. This test can be used to diagnose vitamin D (Vit D) deficiency, and it is particularly indicated in patients with high risk for Vit D deficiency when abnormal calcium, phosphorus, and parathyroid hormone (PTH) levels are observed in a patient (calcium metabolism imbalance), and when the results of the test would be used as supporting evidence for beginning aggressive therapies [1,2,3,4] or supplementation, for example, to boost the immune system [5]. 

From a biochemistry perspective, Vit D is a group of molecules closely connected that derive from a common precursor, 7-dehydrocholesterol or pre-Vit D. In humans, the most important compounds in this group are Vit D2 (ergocalciferol) and D3 (cholecalciferol) [6]. Vit D2 is acquired from food and vitamin supplements, whereas Vit D3 is synthesized by the skin [1,7,8]. Both Vit D2 and Vit D3 are hydroxylated in the liver to produce 25-OH-D, which is further hydroxylated in the kidneys to produce 1,25-(OH)_2_D. Whilst 1,25-(OH)_2_ D2 is the physiologically active form of VD, the total amount of 25-OH-D2 and D3, also known as total 25-OH-D, represents the marker of the overall Vit D status of a patient [9,10,11,12,13]. 

In the context of the general population, the recommended 25-OH-D concentration in adults should fall in the range of 30–50 ng/mL (75–125 nmol/L) [14].

Furthermore, more people are severely deficient in Vit D [25-OH-D < 10 ng/mL, making Vit D measurement very important [15].

Vit D-related scientific studies have grown exponentially over the past decades; as such, nowadays, a large body of scientific literature shows a clear link between Vit D deficiency and various chronic and acute conditions, including bone metabolism disorders, kidney and cardiovascular diseases, obesity, cancer, autoimmune diseases, diabetes, infectious diseases, and aging [16,17]. Accordingly, the demand for accurate Vit D testing in clinical practice, for personalized Vit D supplementation for any health condition, increased more than 10-fold in the past 10 years. A special remark should be made for the critically ill patients admitted to ICU. Vit D deficiency is very common in the ICU because many critically ill patients were already chronically ill before their acute illness. Moreover, as previously mentioned, numerous epidemiological studies link Vit D deficiency to many diseases across a wide variety of organ systems. One of the latest pieces of research on this topic, the VIOLET study, was a randomized controlled, double-blind, placebo-controlled phase III trial conducted with patients with Vit D deficiency and a high risk of developing ARDS (acute respiratory distress syndrome) [18]. According to this large trial, the intake of ordinary doses of Vit D supplements seems to be associated with decreases in total mortality rates, although the relationship between baseline Vit D status, dose of Vit D supplements, and total mortality rates remains to be further investigated. Furthermore, other intensive care unit studies have demonstrated an association between Vit D deficiency [25-OH-D < 20 ng/mL] and increased hospital length of stay (LOS), readmission rate, sepsis, and mortality [18,19]. 

Despite this continuous growing interest in Vit D with consequent increase in requests for determining circulating levels of total 25-OH-D (D2/D3), the analytical performance of these immunoassays, including radioimmunoassay and ELISA, is highly variable, and even mass spectrometry methods, which nowadays serve as the gold standard for the quantitative determination of 25-OH-D, do not necessarily produce comparable results, creating limitations for the definition of normal Vit D status ranges [20]. To solve this problem, great efforts have been made to promote the standardization of laboratory assays, which is important to achieve comparable results across different methods and manufacturers [9,21]. 

In this study, we performed a comparative performance assessment against a gold standard technique/method (LC-MS/MS) of a brand-new, point-of-care, fluorescence immunoassay based on a single cartridge, with a lateral-flow architecture, containing small wells pre-filled with all reagents necessary for the test (so-called “all-in-one” concept). Such a novel test, based on a sandwich immunodetection method, seems to overcome the limits of the current assays available for the measure of Vit D status in the intensive care units and other decentralized scenarios, suggesting it could be widely requested by clinicians, once integrated into a routine, on-site diagnostic service.

One of the best advantages of this method in clinical practice is the fact that point-of-care testing (POCT), performed near patients, generally increases patient satisfaction and involvement without the need for sample transport, decreasing turnaround time (TAT) and by-passing procedure delays [22].

## 2. Results

**Figure 1:**
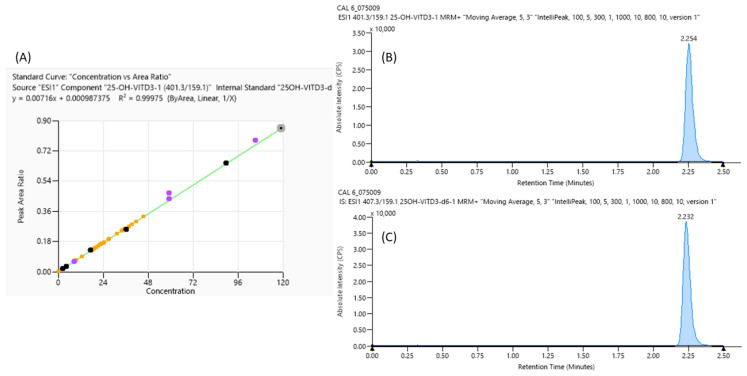
LC-MS/MS Vitamin D analyses: curve calibration parameter (**A**) and mass spectrometry peaks of the highest curve calibrator point of vitamin D (**B**) and its Internal Standard (**C**) with their retention time.

An example of a calibration curve with linearity data (A) and chromatographic peaks of Vit D (B) and its Internal Standard (C) performed with LC-MS/MS, used for the comparative performance assessment, is shown in Figure 1.

Despite 25-OH-D comprising both 25-OH-D2 and 25-OH-D3, the data in the article exclude 25-OH-D2; nevertheless, 25-OH-D2 was not detected in the real samples, probably due to patients’ scarce 25-OH-D2 supplementation or dietary intake. For this reason, no data were reported and only 25-OH-D3 analysis was performed.

In Table 1, it is possible to observe mean and standard deviation values for different methods for fresh, frozen, and thawed samples.

For verifying the homogeneity of variances on AFIAS-1^®^, AFIAS-10^®^, and LC-MS/MS, the Levene non-parametric test was performed and the *p*-values are reported in Table 2. In particular, concerning thawed samples, LC MS/MS showed a homogeneous variance compared to AFIAS-10 (*p* = 0.04).

Following the quantification of Vit D on all the systems (AFIAS-1^®^, AFIAS-10^®^, and LC-MS/MS), the statistical analysis was carried out to evaluate the agreement between two quantitative methods.

Passing–Bablok regression represents a statistical evaluation for nonparametric regression analysis suitable for method comparison studies.

**Figure 2:**
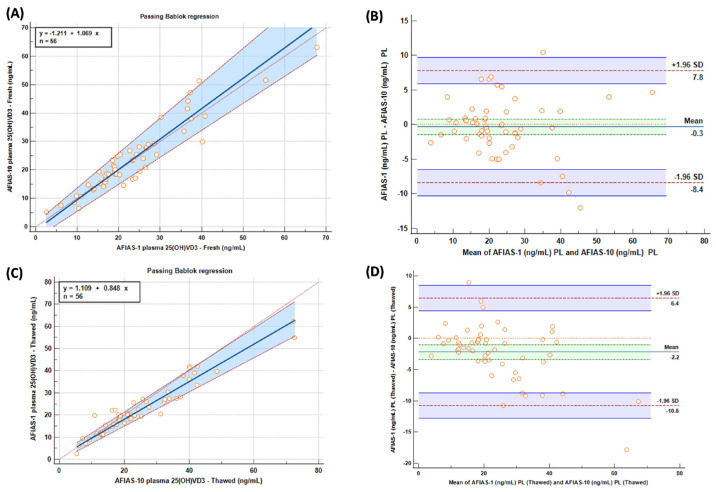
Passing–Bablok (**A**,**C**) and Bland–Altman (**B**,**D**) graphs comparing AFIAS-1 and AFIAS-10 on “fresh” (**A**,**B**) and “frozen” (**C**,**D**) samples.

The Bland–Altman plot is a scatter plot that allows the evaluation of the agreement between two quantitative measurements; the differences between the two measurements (i.e., the measurement error) are shown in Figure 2B,D on the vertical axis, while the horizontal axis represents their arithmetic averages.

In terms of results, Passing–Bablok (A) and Bland–Altman (B) graphs comparing AFIAS-1^®^ and AFIAS-10^®^ on “fresh” and “frozen” (C), (D) samples are shown in Figure 2.

**Figure 3:**
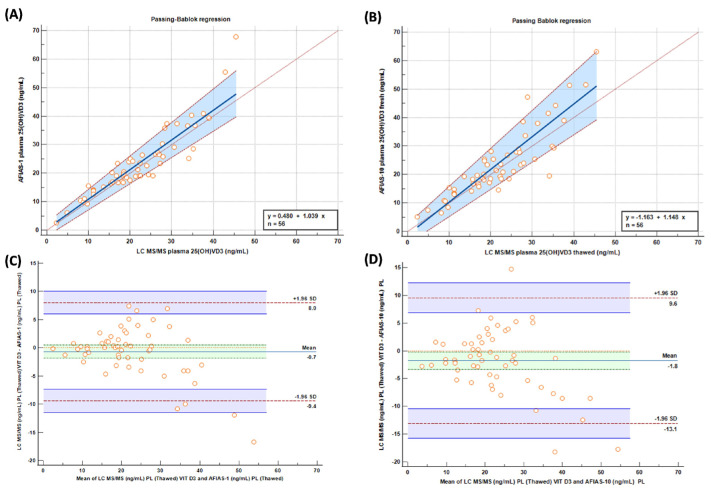
Passing–Bablok and (**A**,**B**) Bland–Altman (**C**,**D**) graphs comparing “fresh” samples tested with AFIAS-1(A) and AFIAS-10(B) with LC-MS/MS method.

Passing–Bablok graphs comparing “fresh” samples tested with AFIAS-1^®^(A) and AFIAS-10^®^(B) with the LC-MS/MS method are reported in Figure 3, and they show a good concordance between the evaluated methods/techniques.

Pearson’s correlation coefficient, investigating LC-MS/MS and AFIAS1 methods, was 0.932 with a *p*-value of 0.0001. Comparing LC-MS/MS with AFIAS 10, Pearson’s coefficient was 0.910 with a *p*-value of 0.0001.

Moreover, we reported the CV% for repeated measurements with different AFIAS methods compared to LC-MS/MS; AFIAS1 compared to LC-MS/MS reported a CV% of 14.2%. AFIAS10 compared to LC-MS/MS reported a CV% of 18.6%.

## 3. Discussion

In recent years, the role of Vit D has been increasingly under the scientific spotlight, suggesting how Vit D supplementation, as an example, might be a useful tool to reduce the risk of infection [19,20], other than bone and calcium homeostasis involvement. Moreover, recent pieces of evidence highlight how this pro-hormone has several activities, such as influencing drug concentrations and regulating the expression of cytochrome P450 genes [23,24,25]. Consequently, Vit D is able to affect drug exposures in different seasons, having an impact on clinical outcome [25].

For all these reasons, quantifying Vit D plasma exposure could represent a useful tool for clinical practice.

This work aims to perform a comparative performance assessment study between AFIAS^®^ fluorescence immunoassay based on a single or multiple cartridge and the gold standard quantitation method LC-MS/MS, and for this reason, the linearity and mass resolution of Vit D quantitation were also verified. In particular, using the Eureka kit, it is possible to observe an excellent linearity in the Vit D quantitation range analyzed, as suggested by the calibration curve and the R^2^ value above 0.999, according to FDA guidelines [26] (Figure 1A). Moreover, the chromatographic peak of Vit D (Figure 1B) and its internal standard are reported in Figure 1C; it is important to highlight how both were eluted at the same retention time, indicating that Internal Standard is a good indicator for this analysis.

Having made these considerations, the samples were also simultaneously analyzed in AFIAS-1^®^ and AFIAS-10^®^ instruments; we have observed a wide percentage of coefficient of variation for all the methods/techniques, showing evidence for the inter-individual variability of 25-OH-VD (Table 1). Nevertheless, all the methods result in around the same median and standard deviation, highlighting how both LC-MS/MS and AFIAS^®^ results are comparable.

In this context, Levene’s test was performed in order to verify whether one method compared to another is able to yield an overestimation of the inter-individual variability (Table 2). 

Delving deeper into this context, the comparison between the LC-MS/MS method and AFIAS-1^®^ showed a *p*-value of 0.4, indicating a likelihood of 40% of having the same variances and 21% considering AFIAS-1^®^ against AFIAS-10^®^; comparing LC-MS/MS results to AFIAS-10^®^, a *p*-value of 0.04 was reported, indicating the likelihood of having an overestimated result, as supported by their CV% in Table 1 (44% against 57%, respectively).

Following a careful comparative statistical investigation, it is possible to observe a very good correlation between AFIAS-1^®^ and AFIAS-10^®^ on ‘fresh’ plasma and a good correlation between AFIAS-1^®^ and AFIAS-10^®^ on ‘frozen’ plasma, with a slight overestimation of AFIAS-10^®^ on the high values, as depicted in the graph in Figure 1. 

Comparing the results obtained with AFIAS-1^®^ on ‘fresh’ plasma with the LC-MS/MS analysis and AFIAS-10^®^ with respect to the LC-MS/MS analysis (Figure 2), it is possible to observe a slight, though not significant, overestimation of AFIAS-10^®^ regarding the high values, supporting the previously mentioned results.

Summarizing, it can be stated that AFIAS-1^®^ seems to perform barely better than AFIAS-10^®^, in comparison with LC-MS/MS analysis; as long as 40 ng/mL of Vit D is not exceeded, then performance seems to deteriorate with a potential lower overestimation. The observed behavior is similar when considering fresh and thawed plasma.

AFIAS-10^®^ shows an acceptable error within 30 ng/mL, but tends to have a rather large proportional deviation. Again, it is possible to observe this behavior in both fresh plasma (approximately +15% proportional overestimation, partially offset at low levels by −1.16 ng/mL intercept) and in thawed plasma (+24% proportional, partially offset at low levels by −2.05 ng/mL intercept).

Moreover, from the results, it is possible to make another consideration; since the two most important decision limits for Vit D deficiency are 10 ng/mL for severe insufficiency and 30 ng/mL for sufficiency [15], it is possible to observe from Bland–Altman analysis (Figure 3) that, at that point, the methods are very comparable with a scarce number of samples outside the limit, with only around 1% of both for AFIAS 1 and AFIAS 10.

Moreover, as suggested by [27], there is a wide variability concerning Vit D intra-individual concentration, and if the intra-individual variability tested was around 12%, the analytical variability must be within 6% concerning the LC/MS-MS method, while concerning the immunometric assay, it is possible to observe how it is inside 12% of the variability.

The bias of this preliminary study is the low number (n = 56) of samples, as this is an exploratory work on a new diagnostic, although it had the opportunity to assess Vit D levels over a wide dynamic range of concentrations.

Concerning the limitations of the study, despite our LC-MS/MS method being able to detect 25(OH)D2, all the measured samples were below the lower limit of detection, probably because patients were not supplemented with 25-OH-D2, but only with 25-OH-D3. In fact, in Italy, there are no clinical recommendations for vitamin D2 supplementation, except for a small sub-population of patients, such as vegans, who cannot obtain vitamin D from foods of animal origin, and therefore, they take vitamin D2 to supplement themselves, which is mainly found in supplements derived from plant sources such as mushrooms [28]. Delving deeper into this context, it could be useful to increase the cohort of samples, also adding patients supplemented with 25-OH-D2, to obtain method validation for ergocalciferol, which was not detectable in the current analyzed cohort.

The advantage of the AFIAS Vitamin Neo All-in-One cartridge is that it avoids the use of reagents or extra equipment, because the user does not need other materials to perform the test. Moreover, the cartridge is constituted of lyophilized granules, making the method stable in a wider range of temperatures. Furthermore, applied in the context of POCTs, this method could be an important tool in order to have quicker analyses, simpler handling of patients and samples, and enhance clinical management of vitamin D deficiency.

## 4. Materials and Methods

This performance assessment study was conducted on AFIAS^®^ Vitamin D Neo test, a fluorescent immunoassay for the quantitative determination of 25-OH-D2/D3 level using AFIAS-1^®^ and AFIAS-10^®^ instrumentation for AFIAS^®^ tests (testing 1 or 10 samples simultaneously), comparing with the gold standard LC-MS/MS technology (A. Menarini Diagnostics s.r.l., 50131 Florence, Italy).

Repeatability, precision, and recovery data concerning AFIAS Vitamin D Neo were previously tested and reported maximum CV% as follows. Measurement repeatability at different Vit D concentrations reached a maximum CV% of 12.9%; within-laboratory precision of 12.63%; and a multi-site study reproducibility of 11.4%. Accuracy in 3 different lots of AFIAS Vitamin D Neo was tested, measuring 10 times at each concentration of the control standard, and none of the recovery values exceeded 105.5%.

A Perkin Elmer LX-50VR UHPLC system coupled with a Triple Quadrupole Q-Sight 220VR (Perkin Elmer, Milan, Italy) as LC-MS/MS was used for the chromatographic analysis using Eureka kit for the sample extraction and determination of Vit D in plasma samples.

The LC-MS/MS Vit D calibrators and quality controls used for the analysis were produced by EUREKA Lab Division (EUREKA srl Lab Division, 60033 Chiaravalle, Italy), according to all the requirements of Directive 98/79/EC concerning in vitro diagnostic medical devices (IVDs), and the declaration of conformity is available upon request.

The EUREKA LC-MS/MS Vit D kit was already tested for its performance in terms of accuracy, precision, and reproducibility by our laboratory (following EMA and FDA guidelines) in a previously published article, and this method was also evaluated for mutual concordance with the IVD COBAS Vit D immunoassay platform [29].

A total of 56 leftover routine blood samples were collected, and the analyses were performed as follows.

All the samples were centrifuged and the plasma was collected; a part of this sample was frozen in 2 distinct aliquots, while the remaining part of the “fresh” plasma was used for the following analyses.

Hence, “fresh” (not frozen) together with the “frozen” plasma analyses were conducted both in AFIAS-1^®^ and AFIAS-10^®^. Moreover, LC-MS/MS plasma analysis was performed on the residue of frozen plasma aliquots, and all analyses have been performed once.

All the statistical analyses were evaluated through the MedCalc calculator (MedCalc Software Ltd, Version 22.014, 8400 Ostend, Belgium, 2023) 

## 5. Conclusions

In conclusion, comparing AFIAS-1^®^ and AFIAS-10^®^ (Boditech & Menarini) results versus LC-MS/MS gold standard, they showed acceptable good agreement, both on fresh and frozen plasma samples, with a lower, but non-clinically relevant, overestimation, particularly for AFIAS-10^®^, at very high levels of 25-OH-VD.

In the most sensitive and clinically relevant range (between 10 and 35 ng/mL), we have observed a good statistical correlation between AFIAS-1^®^ and AFIAS-10^®^ results versus LC-MS/MS analyses.

## Figures and Tables

**Table 1:** Mean vitamin D concentrations and inter-individual variability (as standard deviations and CV%) observed in our sample population.

	AFIAS-1 (ng/mL)	AFIAS-10 (ng/mL)	AFIAS-1 (ng/mL)-Thawed	AFIAS-10 (ng/mL)-Thawed	LC MS/MS (ng/mL)-Thawed
**Vitamin D3** **(mean ± SD)**	23.37 ± 11.53	23.69 ± 12.00	22.61 ± 11.72	24.79 ± 14.16	21.92 ± 9.64
**CV%**	49%	51%	52%	57%	44%

**Table 2:** Levene test *p*-value between LC-MS/MS, AFIAS-1, and AFIAS-10.

	AFIAS-1 (ng/mL)-Thawed	AFIAS-10 (ng/mL)-Thawed
**LC MS/MS (ng/mL)-Thawed**	0.40	0.04
**AFIAS-1 (ng/mL)-Thawed**	-	0.21

## Data Availability

Data are available upon request.
